# Oxygen-Tolerant Inverse Microemulsion and Miniemulsion PhotoATRP

**DOI:** 10.1021/acsmacrolett.5c00825

**Published:** 2026-01-16

**Authors:** Xiaolei Hu, Rongguan Yin, Krzysztof Matyjaszewski

**Affiliations:** Department of Chemistry, 6612 Carnegie Mellon University, Pittsburgh, Pennsylvania 15213, United States

## Abstract

Reversible deactivation radical polymerization (RDRP) in an emulsion is a practical and environmentally friendly route to well-defined polymer synthesis. However, most emulsion RDRP has focused on conventional oil-in-water systems, restricting accessible materials to hydrophobic polymers. Here, we report the first example of a highly efficient and oxygen-tolerant inverse microemulsion and miniemulsion photoinduced ATRP (photoATRP) facilitated by a dual catalytic system. Irradiation with red light efficiently excites the photocatalyst methylene blue (MB^+^), facilitating the photoreduction of the deactivator to initiate and mediate polymerization. This process enables the precise synthesis of polymers with a controlled molecular weight, low dispersity (*Đ* ≤ 1.20), excellent chain-end fidelity, and temporal control. The versatility of this approach was further demonstrated by expanding the photocatalyst scope beyond MB^+^ to include a library of other water-soluble PC. This method was also successfully extended to the inverse miniemulsion. This work establishes a practical inverse emulsion photoATRP for synthesizing well-defined hydrophilic polymers.

## Graphical abstract

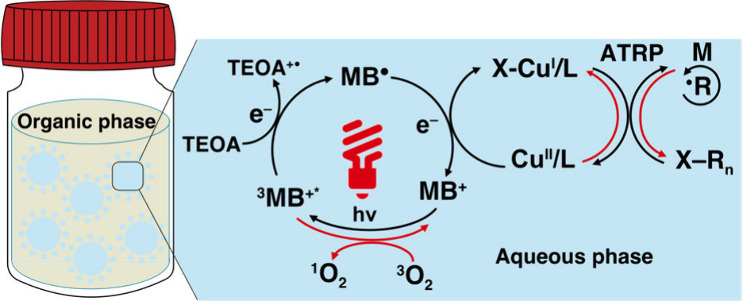

Emulsion polymerization is widely adopted in industrial polymer manufacturing due to its excellent heat transfer, enhanced control over reaction kinetics, reduced amount of toxic organic solvents, and ability to produce high-molecular-weight polymers at low overall viscosity.^1–8^ Traditionally performed via free-radical polymerization (FRP), this method suffers from uncontrolled radical propagation and irreversible termination, resulting in broad molecular weight distributions and limited architectural control. Implementing reversible deactivation radical polymerization (RDRP) into emulsion overcomes these intrinsic limitations, enabling precise control over molecular weight, dispersity, end-group fidelity, sequence, and macromolecular architecture.^9–21^ These advances have significantly broadened the applicability of emulsion polymerization in material science and biomedicine.^22,23^

Most RDRPs in emulsions have focused on conventional oil-in-water (O/W) systems, where polymerization occurs within hydrophobic monomer droplets or polymer particles dispersed in an aqueous phase. Consequently, the products are primarily limited to hydrophobic polymers.^22^ In contrast, inverse emulsion polymerization, a water-in-oil (W/O) system, addresses this limitation by polymerizing hydrophilic monomers within nanoscale aqueous droplets dispersed in an organic continuous phase. This strategy enables controlled synthesis of diverse water-soluble polymers with high molecular weight that are widely used as flocculants, stabilizers, absorbents, and viscosity modifiers. Furthermore, inverse emulsion is a robust approach for producing functional nanogels with tunable size and narrow size distribution, which have found diverse biomedical applications.^24–26^

Efforts to implement RDRP in inverse emulsion have primarily involved microemulsion and miniemulsion systems.^27,28^ For instance, atom transfer radical polymerization (ATRP) in inverse microemulsion was initiated through in situ generation of the [Cu^I^/ligand]^+^ activator using reducing agents.^29^ Alternatively, the ATRP process was also initiated by UV light through either the use of photoinitiators or the direct photoreduction of the ATRP deactivator.^27^ Reversible addition–fragmentation chain transfer (RAFT) polymerizations have also been investigated by directly generating propagating radicals through a photoiniferter or a photoinduced electron/energy transfer (PET) process from an excited photosensitizer under UV.^30–32^ Despite these advances, the inverse emulsion RDRP systems have notable limitations, including the slow polymerization rate, poor O_2_ tolerance, and predominant use of UV and short-wavelength light. Recent years have seen significant advancements in the development of oxygen-tolerant RDRP.^33–36^ For example, our group recently reported highly efficient and O_2_-tolerant ATRP and RAFT processes in homogeneous and heterogeneous media using a red/NIR light-active photocatalyst (PC), methylene blue (MB^+^).^10,36–39^ The PC, excited under red/NIR light, is advantageous owing to the high photocatalytic efficiency, efficient oxygen quenching, enhanced light penetration, and less destructive characteristics.^40–43^ We anticipate that these advantages of MB^+^ could address the persistent challenges and facilitate RDRP in the inverse emulsion.

Herein, we report the first fully oxygen-tolerant photoinduced ATRP (photoATRP) carried out in an inverse microemulsion and miniemulsion using a dual-catalytic system composed of water-soluble MB^+^ and Cu/TPMA (TPMA = tris­(2-pyridylmethyl)­amine) complexes. As illustrated in Scheme 1, the aqueous ATRP phase was dispersed and stabilized within an organic continuous phase with the aid of mixed surfactants, enabling rapid and well-controlled polymerization inside nanodroplets without prior deoxygenation. This platform affords hydrophilic polymers and block copolymers with excellent molecular weight control, chain-end fidelity, and temporal regulation. The versatility of this approach was further demonstrated by expanding the photocatalyst scope beyond MB^+^ to include a library of other water-soluble PCs. Additionally, it was further extended to an inverse miniemulsion with less surfactants to produce larger polymer particles. The robust inverse emulsion photoATRP represents an advance in controlled polymerization of water-soluble monomers and provides a versatile route to well-defined hydrophilic polymers and functional nanomaterials for biomedical and other advanced applications.

**Scheme 1:**
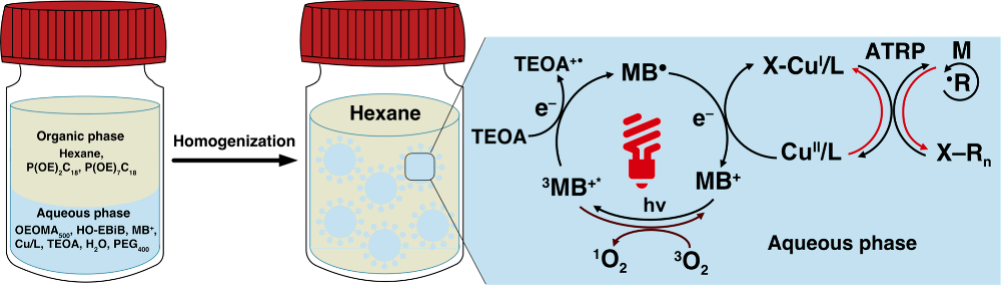
Inverse Microemulsion PhotoATRP Mediated by MB^+^ and CuBr_2_/TPMA

An inverse microemulsion is fundamentally a nanoscale aqueous phase dispersed in a continuous organic phase and stabilized by the surfactant (Scheme 1). To validate the feasibility of our concept for inverse emulsion photoATRP, we first adapted the aqueous photoATRP conditions from a previously established homogeneous aqueous photoATRP system.^36^ The aqueous ATRP phase consists of oligo­(ethylene oxide) methyl ether methacrylate (OEOMA_500_, average *M*_n_ = 500) as the monomer, 2-hydroxyethyl α-bromoisobutyrate (HO–EBiB) as the initiator, MB^+^ as the photocatalyst, [X–Cu^II^/TPMA]^+^ complex as the deactivator, and triethanolamine (TEOA) as the electron donor (ED) in phosphate-buffered saline (PBS) solution. Stabilization of this aqueous phase in organic media is critical for achieving a successful inverse emulsion. The results showed that a surfactant mixture of polyoxyethylene oleyl ethers (P­(EO)_*n*_C_18_, *n* = 2 and 7), combined with PEG_400_ as a stabilizer and hexane as the continuous organic phase, yielded the most stable inverse microemulsion after vortexing (Figure S1, P­(EO)_2_C_18_/P­(EO)_7_C_18_/PEG_400_/hexane = 0.2/0.4/0.17/5, wt/wt).^29^ This formulation aligns with previous inverse emulsion RDRP systems, attributed to the optimal hydrophilic–lipophilic balance (HLB) for W/O emulsion achieved by mixed surfactants.^29,32^ Upon red light irradiation (640 nm) of the resulting inverse microemulsion for 1 h without any prior deoxygenation, rapid polymerization occurred with a monomer conversion of 93% (Table 1, entry 1) and good control over molecular weight and low dispersity (*M*_n,abs_ = 24800, *M*_n,th_ = 23300, *Đ* = 1.12; Figure S2). Dynamic light scattering (DLS) analysis of the translucent emulsion revealed an average particle diameter (*Z*_avg_) of 72.5 nm (Figure S3), consistent with a typical inverse microemulsion polymerization. To elucidate the mechanism, control experiments were conducted. Similar to the homogeneous polymerization, the exclusion of the photocatalyst MB (Table 1, entry 2) or ATRP deactivator (Table 1, entry 3) caused no monomer conversion. These results confirm that the inverse emulsion photoATRP mediated by MB^+^/Cu follows the mechanism established in aqueous photoATRP.^36^

**Table 1:** Optimization of Inverse Microemulsion PhotoATRP Conditions^a^

entry	HO-EBiB (equiv)	MB^+^ (equiv)	CuBr_2_/L (equiv)	time (h)	conv.^b^ (%)	*M*_n,th_	*M*_n,app_ ^c^	*M*_n,abs_ ^d^	*Đ* ^c^	*Z*_avg_ ^e^ (nm)
1	1	0.025	0.15	1	93	23300	23700	24800	1.12	72.5 ± 0.4
2	1		0.15	1	3					
3	1	0.025		1	2					
4	2	0.025	0.15	1	93.8	11718	16170	15600	1.11	68.9 ± 0.2
5	0.5	0.025	0.15	2.5	99	50000	34500	39100	1.11	59.1 ± 0.2
6^f^	0.5	0.025	0.15	1.5	99	50000	29000	31660	1.09	127 ± 0.7

a Standard reaction conditions: [OEOMA_500_]/[HO-EBiB]/[MB^+^]/[CuBr_2_/L]/[TEOA] = 50/X/X/X/0.3, OEOMA_500_/P­(EO)_2_C_18_/P­(EO)_7_C_18_/PEG_400_/hexane = 0.25/0.2/0.4/0.17/5 by weight (g). [OEOMA_500_] = 4 wt % to total, [P­(EO)_*n*_C_18_] = 9.6 wt % to total. Reactions were irradiated under red LED (640 nm, 25 mW cm^–2^) in a one-dram vial (5 mL, diameter = 15 mm) with stirring (700 rpm).

b Monomer conversion was determined by ^1^H NMR with internal standard DMF.

c Apparent molecular weight (*M*_n,app_) and dispersity (*Đ*) were determined by SEC analysis (DMF as eluent) calibrated to PMMA standards.

d Absolute molecular weight (*M*_n,abs_) was determined by Mark–Houwink calibration.

e Average particle diameter (*Z*_avg_) was determined by DLS.

f Lower surfactant concentration of 7.2 wt % relative to total.

We then examined the capability of inverse microemulsion photoATRP to regulate the molecular weight (MW) of the resulting polymers. By varying the initiator concentration while maintaining constant concentrations of other components, polymers with various degrees of polymerization (DP_T_) ranging from 25 to 100 were targeted (Table 1, entries 1, 4, and 5). In all cases, monomer conversion reached >90%, yielding polymers with controlled MW and low dispersity (*Đ* ≤ 1.12).

Surfactant concentration plays a critical role in both the colloidal stability and kinetics. When decreasing the concentration of surfactant from 9.6 to 7.2 wt % relative to the total mixture, it increased particle size from 72.5 to 127 nm (Table 1, entry 6, Figure S4). Additionally, at a lower surfactant concentration, deviation between *M*_n,abs_ and *M*_n,th_ became more pronounced, likely due to the side reactions associated with reduced stability. A further decrease in surfactant concentration (e.g., ≤4.8%) caused macroscopic phase separation during polymerization.

Given the broad absorption profile of MB^+^ and its demonstrated efficacy across multiple wavelengths in homogeneous and miniemulsion photoATRP,^10,36^ we investigated the capability of conducting inverse photoATRP under other light wavelengths (UV, blue, green, and NIR lights). As expected, the inverse emulsion proceeded successfully from 390 to 740 nm with good MW control and low dispersity (Table S1).

The kinetic analysis of the inverse microemulsion revealed a short induction period of ca. 20 min (Figure 1A), corresponding to the time required for O_2_ consumption in the reaction mixture. Following this induction phase, polymerization proceeded rapidly, reaching nearly quantitative monomer conversion within 60 min. The slightly longer induction period compared to aqueous photoATRP is likely attributed to the higher O_2_ concentration in the continuous organic phase (hexane) compared to water, as well as a slower O_2_ quenching rate due to the lower [MB^+^] in the inverse emulsion.^44,45^ The absolute molecular weights (*M*_n,abs_) of pOEOMA_500_ increased linearly with monomer conversion and showed an agreement with the theoretical values (*M*_n,th_; solid line in Figure 1B), while maintaining narrow molecular weight distributions (*Đ* ≤ 1.20). In addition, the monomodal SEC traces shifted toward the higher MW region with increased irradiation time (Figure 1C). These results demonstrated that an efficient and well-controlled photoATRP inverse microemulsion was achieved using MB^+^ without prior deoxygenation.

**Figure 1:**
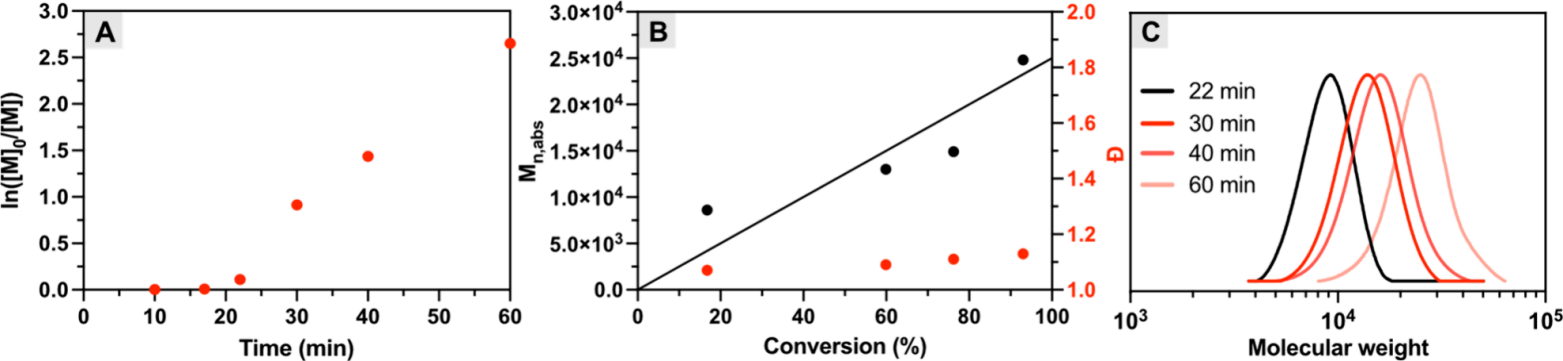
Inverse microemulsion photoATRP under red light. (A) First-order kinetic plot. (B) Evolution of molecular weight and dispersity with monomer conversion. (C) SEC traces evolution over time. Reaction conditions: Standard reaction conditions: [OEOMA_500_]/[HO-EBiB]/[MB^+^]/[CuBr_2_/TPMA]/[TEOA] = 50/1/0.025/0.15/0.3. OEOMA_500_/P­(EO)_2_C_18_/P­(EO)_7_C_18_/PEG_400_/hexane = 0.25/0.2/0.4/0.17/5 by weight (g), irradiated under red LED (640 nm, 25 mW cm^–2^) in a one-dram vial (5 mL, diameter = 15 mm) with stirring (700 rpm).

The chain-end fidelity of polymers synthesized by inverse microemulsion photoATRP was examined through chain extension experiments (Figure 2A). First, pOEOMA_500_ was synthesized by inverse microemulsion photoATRP (*M*_n,abs_ = 13700, *Đ* = 1.10) and subsequently employed as a macroinitiator for chain extension with OEOMA_500_. The SEC trace of the resulting block copolymer (pOEOMA_500_-*b*-pOEOMA_500_) shifted to the higher MW region without tailing or a shoulder peak (*M*_n,abs_ = 41400, *Đ* = 1.14). The corresponding inverse microemulsion exhibited a uniform particle size distribution (*Z*_avg_ = 82 nm, Figure S5). A successful chain extension was achieved using the hydrophilic macroinitiator PEG_2k_-Br (Figures 2B and S6), yielding a well-defined block copolymer PEG-*b*-pOEOMA_500_ (*M*_n,app_ = 16900, *Đ* = 1.12; *Z*_avg_ = 65 nm). These results confirm that inverse microemulsion photoATRP proceeds in a well-controlled manner, effectively suppressing undesired terminations of polymer chains.

**Figure 2:**
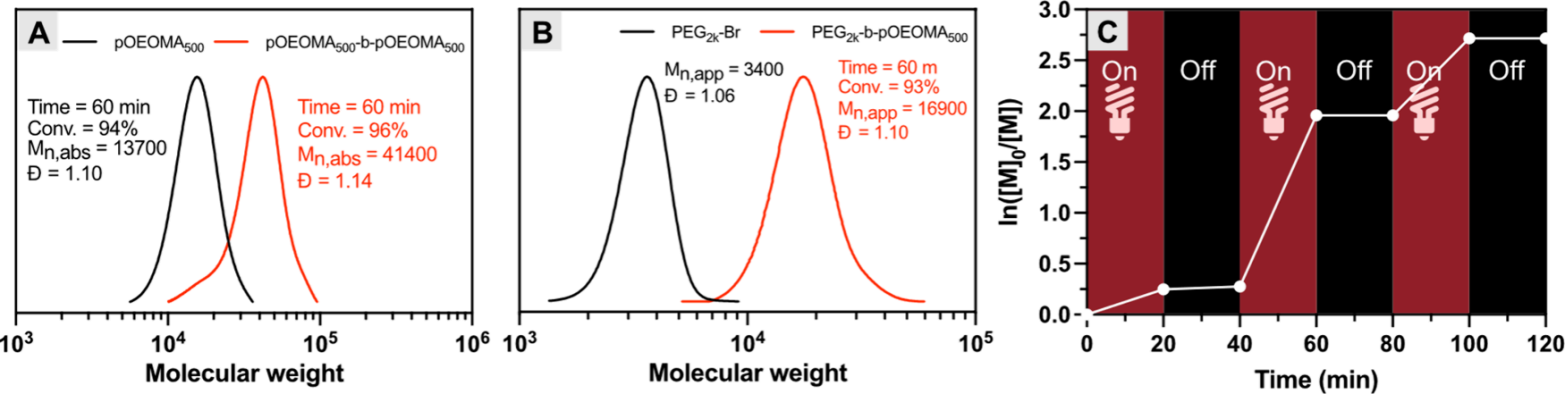
Chain extension of (A) pOEOMA_500_ and (B) PEG_2k_-Br macroinitiators with OEOMA_500_. (C) Temporal control of inverse microemulsion photoATRP under red LED in a one-dram vial with stirring.

In addition, the photoinduced polymerization enables temporal control by modulating light exposure. When the light was switched off, negligible monomer conversion was observed (Figure 2C). Upon irradiation with red light, photoinduced regeneration of the ATRP activator by excited MB^+^ reinitiated the polymerization. Several on/off irradiation cycles were successfully performed, demonstrating excellent temporal control. The resulting polymer showed a general agreement between *M*_n,abs_ and the theoretical value (*M*_n,th_ = 11700, *M*_n,abs_ = 13400) and maintained a low dispersity (*Đ* = 1.11, Figure S7A). DLS measurements reveal a monodistributed particle size of 55.2 nm (Figure S7B).

One of the fundamental challenges of the previous inverse emulsion photoRDRP stems from poor oxygen tolerance and inefficient photocatalysis. These limitations not only led to slow initiation and propagation but also required tedious deoxygenation steps prior to polymerization. Recently, in addition to MB^+^, several water-soluble PCs have been reported to enable O_2_-tolerant photoATRP in both aqueous and organic media through dual catalytic mechanism.^46^ We envisioned that these PCs could also enhance photoATRP in an inverse emulsion. Therefore, inverse microemulsion polymerizations were conducted using several PCs, including eosin Y (EY), rose bengal (RB), rhodamine 6G (RD-6G), and rhodamine B (RD), under the same reaction conditions already established for MB^+^ (Table 2). Consistent with results in homogeneous polymerization, these excited PCs under green light successfully mediated polymerization (*Đ* < 1.20), yielding polymer particles with monomodal size distributions ranging from 70 to 180 nm. Interestingly, polymerization mediated by MB^+^ under red light was the most efficient (Table 2, entry 1) among the five photocatalysts tested under identical conditions. This enhanced performance is likely attributed to the high catalytic efficiency of MB^+^/Cu dual catalytic system under longer wavelength light, which allows deeper light penetration for facilitating emulsion polymerization.^10^

**Table 2:**
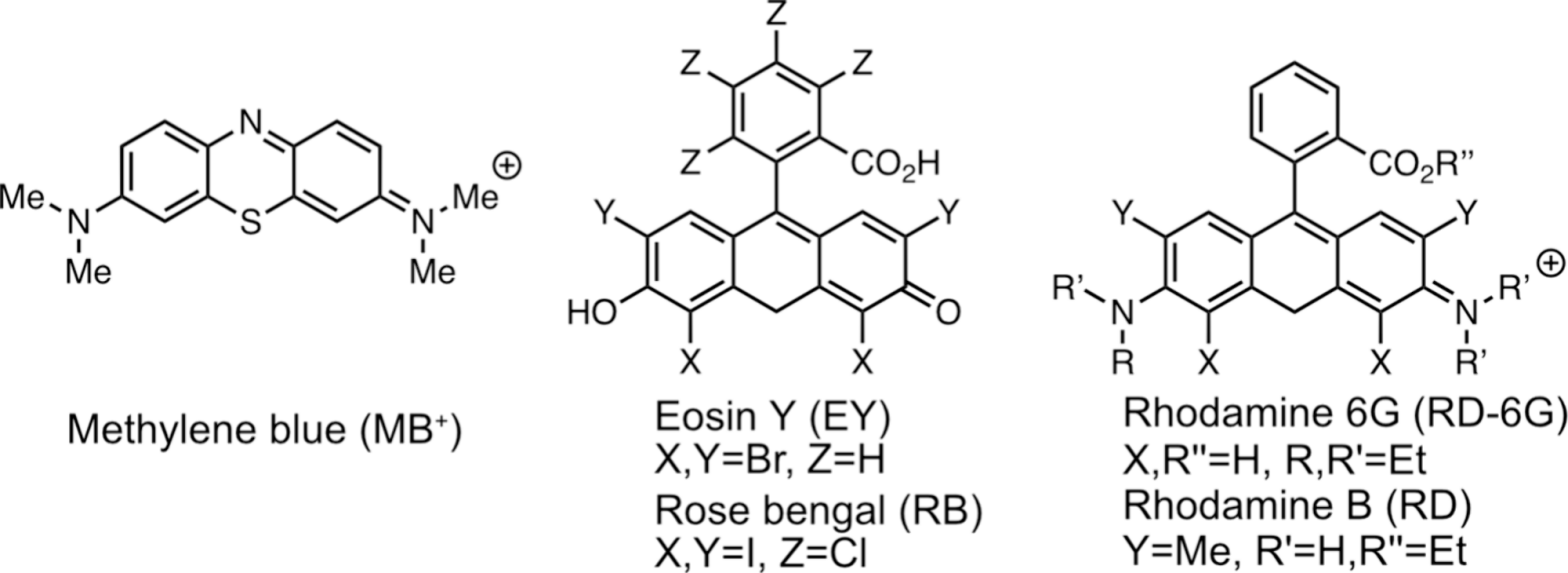
Inverse Microemulsion PHOTOATRP of OEOMA_500_ Using PCs^a^

entry	PC	λ_max_ (nm)	intensity (mW cm^–2^)	time (h)	conv.^b^ (%)	*M*_n,th_	*M*_n,abs_ ^c^	*Đ* ^d^	*Z*_avg_ ^e^ (nm)
1	MB	640	25	1	93	23300	24800	1.12	72.5 ± 0.4
2	EY	520	25	1	0				
3	EY	520	25	4	98	24700	17800	1.15	80 ± 0.1
4	RB	520	25	1	0				
5	RB	520	25	4	99	24500	18400	1.18	87 ± 0.4
6	RD-6G	520	25	4	0				
7	RD-6G	520	25	12	79	19700	16900	1.12	109 ± 0.3
8	RD	520	25	12	5				
9	RD	520	50	12	92	23018	13900	1.10	172 ± 0.3

a Standard reaction conditions: [OEOMA_500_]/[HO-EBiB]/[PC]/[CuBr_2_/TPMA]/[TEOA] = 50/1/0.025/0.15/0.3. OEOMA_500_/P­(EO)_2_C_18_/P­(EO)_7_C_18_/PEG_400_/hexane = 0.25/0.2/0.4/0.17/5 by weight (g), irradiated under different in a one-dram vial (5 mL, diameter = 15 mm) with stirring (700 rpm).

b Monomer conversion was determined by ^1^H NMR with internal standard DMF.

c Absolute molecular weight (*M*_n,abs_) was determined by Mark–Houwink calibration.

d Dispersity (*Đ*) was determined by SEC analysis (DMF as eluent) calibrated to PMMA standards.

e Average particle diameter (*Z*_avg_) was determined by DLS.

Inverse miniemulsion is another widely explored W/O system for the synthesis of hydrophilic polymers and nanoparticles.^47^ To further demonstrate the robustness of our approach, we extended it to inverse miniemulsion by dispersing the aqueous ATRP phase in cyclohexane with the surfactant Span80 (4.3 wt % relative to the total) followed by sonication. After red-light irradiation for 1 h, the polymerization reached 66% monomer conversion with controlled MW and low dispersity (*M*_n,abs_ = 25800, *M*_n,th_ = 32800, *Đ* = 1.16; Figure 3A). The resulting particles had an average size of 415 nm (Figure 3B), which is within the typical range for an inverse miniemulsion and is larger than particles obtained from microemulsion. These results, together with the microemulsion system, highlight the versatility and robustness of our strategy for developing O_2_-tolerant photoATRP in inverse emulsion.

**Figure 3:**
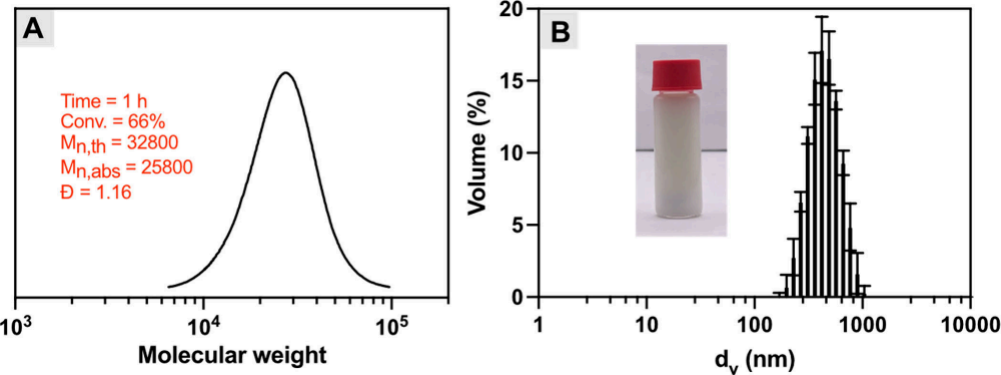
Inverse miniemulsion photoATRP under red light. (A) SEC trace and (B) DLS results with a digital image (inset) of the resulting inverse miniemulsion. ^a^Standard reaction conditions: [OEOMA_500_]/[HO-EBiB]/[MB^+^]/[CuBr_2_/TPMA]/[TEOA] = 100/1/0.025/0.3/0.6, and OEOMA_500_/ Span80/cyclohexane = 0.25/0.25/5 by weight (g), irradiated under red LED (640 nm, 25 mW cm^–2^) in a one-dram vial (5 mL, diameter = 15 mm) with stirring (700 rpm).

In this work, we report the first example of a highly efficient and fully tolerant of the O_2_-tolerant inverse microemulsion and miniemulsion photoATRP facilitated by a dual catalytic system composed of MB^+^ and Cu/TPMA complexes. The excitation of MB^+^ by red light enabled rapid and well-controlled ATRP within nanoscale aqueous droplets dispersed in an organic continuous phase without any deoxygenation steps. The polymerizations yielded polymers with controlled molecular weights, low dispersities (*Đ* ≤ 1.20), excellent chain-end fidelity, and robust temporal control, demonstrating characteristic behavior of a controlled polymerization. The versatility of this approach was further demonstrated by expanding the photocatalyst scope beyond MB^+^ to a library of other water-soluble PCs (EY, RB, RD-6G, and RD), all of which successfully mediated inverse microemulsion polymerizations. Notably, MB^+^ under red-light irradiation produced the fastest polymerizations and better MW control, highlighting the advantages of long-wavelength photocatalyst MB^+^ for heterogeneous polymerization. The method was also extended to inverse miniemulsion, yielding larger polymer particles (∼415 nm) while maintaining good control over the molecular weight and dispersity. Collectively, these results establish a robust and O_2_-tolerant inverse micro/miniemulsion photoATRP platform capable of synthesizing well-defined hydrophilic polymers, block copolymers, and nanoparticles with potential applications in drug delivery, bioconjugation, and water treatment.

## Supplementary Material



## Notes

Additional details of experimental procedures and results, including SEC traces and DLS results (PDF)
