# Supplementary material for: Oxygen-Tolerant Inverse Microemulsion and Miniemulsion PhotoATRP
Source: ACS Macro Lett. 2026 Jan 16;15(2):316–21. doi: 10.1021/acsmacrolett.5c00825 (PMC12918716; doi:10.1021/acsmacrolett.5c00825)
Supplement: Supplementary file 1 [file mz5c00825_si_001.pdf]

Supporting Information

Oxygen-Tolerant Inverse Microemulsion and Miniemulsion PhotoATRP

Xiaolei Hu, Rongguan Yin, and Krzysztof Matyjaszewski*

Department of Chemistry, Carnegie Mellon University, Pittsburgh, Pennsylvania 15213, USA

*Correspondence: K.M. (km3b@andrew.cmu.edu)

Table of Contents

Materials and Instruments S3

Materials S3

Instruments S3

Experimental Procedures S3

General procedure for inverse microemulsion photoATRP of $OEOMA_{500}$ S3

Synthesis of $pOEOMA_{500}$ with varying DP_T S4

Chain extension S4

Temporal control S4

Inverse microemulsion photoATRP using different photocatalysts S5

Inverse miniemulsion photoATRP under red light S5

Supplementary figures and tables S6

References S10

Materials and Instruments

Materials

Unless otherwise noted, all chemicals were purchased from commercial suppliers and used as received. Methylene blue (MB^+, 99%), copper(II) bromide ($CuBr_2$, 99.99%), oligo(ethylene glycol) methyl ether methacrylate (average M_n = 500, $OEOMA_{500}$), sodium bromide (NaBr, ≥99%), triethanolamine (TEOA, ≥99%), poly(ethylene glycol) (average M_n = 500, PEG_{400}, 99%), and span80 were purchased from *Sigma-Aldrich*. Polyethylene glycol monooleyl ethers ($HO(CH_2CH_2O)_nC_{18}H_{35}$, n=approx. 2 or 7) and 2-hydroxyethyl α-bromoisobutyrate (HO-EBiB, 95%) were purchased from TCI Chemicals. All monomers were passed through a column of basic alumina to remove the inhibitor before use. Tris(2-pyridylmethyl)amine (TPMA, 99%) was purchased from *AmBeed*. 10X phosphate-buffered saline (10X PBS) was purchased from *Thermo Fisher Scientific*. Water (HPLC grade), dimethylformamide (DMF, HPLC grade), tetrahydrofuran (THF, HPLC grade), hexane (HPLC grade), cyclohexane (HPLC grade), and dimethyl sulfoxide (DMSO, HPLC grade) were purchased from *Fisher Chemical*. D_2O and DMSO-d_6 was purchased from *Cambridge Isotope Laboratories, Inc.*

Instruments

Polymerization was conducted in a EvoluChem™ PhotoRedOx Box purchased from *Hepatochem* with varying LEDs. LEDs with UV light (390 nm, 25 mW cm^{-2}), green light (520 nm, 25 mW cm^{-2}), and red light (640 nm, 25 mW cm^{-2}) were purchased from *Kessil* except for NIR light (740 nm, 20 mW cm^{-2}) from *Hepatochem.* Size exclusion chromatography (SEC) measurements of polymers were performed using *PSS* columns (Styrogel 10^5, 10^3, 10^2 Å) with DMF containing LiBr (0.05 M) as the eluent at 50 °C and the flow rate of 1 mL/min. Linear PMMA standards were used for SEC calibration. Absolute molecular weight ($M_{n,abs}$) was determined by Mark-Houwink calibration.[1] Ultrasound treatment to prepare miniemulsion samples was carried out using Autotune Series High Intensity Ultrasonic Processor, 1500-Watt Model. Particle sizes were determined by dynamic light scattering (DLS) using a Zetasizer Nano from *Malvern Instruments, Ltd*. ^{1}H Nuclear magnetic resonance (^{1}H NMR) was recorded on *Bruker Avance III* 500 MHz spectrometer for determining monomer conversion during inverse emulsion photoATRP using DMF as the internal standard.

Experimental Procedures

General procedure for inverse microemulsion photoATRP of $OEOMA_{500}$

Stock solutions of HO-EBiB (100 mM in H_2O), $CuBr_2$/TPMA complex (1:1 molar ratio, 50 mM in H_2O), MB^+ (1.88 mM in H_2O), and TEOA (100 mM in H_2O) were prepared. A typical aqueous ATRP phase was prepared by mixing $OEOMA_{500}$ (231.5 µL), HO-EBiB (50 µL), MB^+ stock (133.7 µL), $CuBr_2$/TPMA stock (30 µL), TEOA stock (30 µL), PBS stock (83.5 µL), DMF (25 µL), PEG_{400} (0.17 g), and H_2O (250 µL). The organic phase was prepared by mixing

$P(EO)_2C_{18}$ (0.2 g) and $P(EO)_7C_{18}$ (0.4 g) with hexane (5 g). The prepared ATRP solution was added to the organic phase, and the mixture was homogenized by vortexing for 1 min. The resulting inverse emulsion was placed in a one-dram vial (15 mm diameter) equipped with magnetic stirring and irradiated in an EvoluChem™ PhotoRedOx Box under red LEDs (640 nm, 25 mW cm^{-2}) for 60 min with stirring at 700 rpm. Aliquots were withdrawn periodically during the polymerization for ^1H NMR and SEC analysis. For wavelength-dependent studies, samples were irradiated using UV (390 nm, 25 mW cm^{-2}), green (520 nm, 25 mW cm^{-2}), or NIR (740 nm, 20 mW cm^{-2}) light under otherwise identical conditions.

Synthesis of $pOEOMA_{500}$ with varying DP_T

The target degree of polymerization (DP_T = 25, 50, and 100) was varied by adjusting the [HO-EBiB] while keeping the other components at the same concentration ($[OEOMA_{500}]/[HO\text{-}EBiB]/[MB^+]/[CuBr_2/TPMA]/[TEOA]$ = 50/x/0.025/0.15/0.3, where x = 0.5, 1, or 2). Inverse emulsion photoATRP mixtures were prepared following the general procedure and transferred to a one-dram vial (diameter = 15 mm) equipped with a magnetic stirring bar. Polymerizations were conducted under red LED irradiation (640 nm, 25 mW cm^{-2}) under stirring (700 rpm). At the end of the polymerizations, samples were collected for ^1H NMR and SEC analysis.

Chain extension

For the synthesis of $pOEOMA_{500}$-*b*-$pOEOMA_{500}$ block copolymer, $pOEOMA_{500}$ with DP_T = 25 was first synthesized using molar ratios of $[OEOMA_{500}]/[HO\text{-}EBiB]/[MB^+]/[CuBr_2/TPMA]/[TEOA]$ = 50/2/0.025/0.15/0.3 and irradiated by red LED (640 nm, 25 mW cm^{-2}) under stirring (700 rpm). The crude macroinitiator sample $pOEOMA_{500}$ obtained directly after polymerization was then used without purification to prepare the ATRP mixture for chain extension with $OEOMA_{500}$ at DP_T = 125 under $[OEOMA_{500}]/[pOEOMA_{500}]/[MB^+]/[CuBr_2/TPMA]/[TEOA]$ = 50/0.4/0.025/0.15/0.3. The polymerization mixture was irradiated under red LED irradiation (640 nm, 25 mW cm^{-2}) under stirring (700 rpm). Finally, the sample was withdrawn for ^1H NMR and SEC measurement.

For the synthesis of PEG_{2k}-*b*-$pOEOMA_{500}$ block copolymer, PEG_{2k}-Br macroinitiator was used to prepare the ATRP mixture for chain extension with $OEOMA_{500}$ at DP_T = 25 under the condition of $[OEOMA_{500}]/[PEG_{2k}\text{-}Br]/[MB^+]/[CuBr_2/TPMA]/[TEOA]$ = 50/2/0.025/0.15/0.3. Polymerization was conducted under red LED irradiation (640 nm, 25 mW cm^{-2}) under stirring (700 rpm). At the end of the reactions, samples were collected for ^1H NMR and SEC analysis.

Temporal control

The inverse microemulsion ATRP mixture (5 mL) was prepared according to the general procedure at $[OEOMA_{500}]/[HO\text{-}EBiB]/[PC]/[CuBr_2/TPMA]/[TEOA]$ = 50/2/0.025/0.15/0.3. The polymerization mixtures were irradiated under red LED (640 nm, 25 mW cm^{-2}) under stirring (700 rpm). The light was switched on/off periodically every 10 min and 100 μL of samples were withdrawn at each time point for ^1H NMR and SEC measurement.

Inverse microemulsion photoATRP using different photocatalysts

Polymerizations using different PC were conducted by replacing MB^+ with EY, RB, RD-6G, or RD while maintaining identical component ratios ([$OEOMA_{500}$]/[HO-EBiB]/[PC]/[$CuBr_2$/TPMA]/[TEOA] = 50/1/0.025/0.15/0.3. Inverse emulsion photoATRP mixtures were prepared following the general procedure and transferred to a one-dram vial (diameter = 15 mm) equipped with a magnetic stirring bar. Polymerizations were conducted under green LED irradiation (520 nm) under stirring (700 rpm). At the end of the polymerizations, samples were collected for ^1H NMR and SEC analysis.

Inverse miniemulsion photoATRP under red light

Stock solutions of HO-EBiB (100 mM in H_2O), $CuBr_2$/TPMA complex (1:1 molar ratio, 50 mM in H_2O), MB^+ (1.88 mM in H_2O), and TEOA (100 mM in H_2O) were prepared. A typical aqueous ATRP phase was prepared by mixing $OEOMA_{500}$ (231.5 µL), HO-EBiB (50 µL), MB^+ stock (133.7 µL), $CuBr_2$/TPMA stock solution (30 µL), TEOA stock solution (30 µL), PBS stock (83.5 µL), DMF (25 µL), and H_2O (250 µL). The organic phase was prepared by mixing Span80 (0.25 g) with cyclohexane (5 g). The prepared ATRP solution was added to the organic phase, and the mixture was homogenized by a probe sonication (amplitude = 25%, application and rest time of 1 s each) for 10 min under an ice-water bath. The resulting inverse miniemulsion was placed in a one-dram vial (15 mm diameter) equipped with magnetic stirring and irradiated in an EvoluChem™ PhotoRedOx Box under red LEDs (640 nm, 25 mW cm^{-2}) for 60 min with stirring at 700 rpm. Aliquots were withdrawn periodically during the polymerization for ^1H NMR and SEC analysis.

Supplementary figures and tables

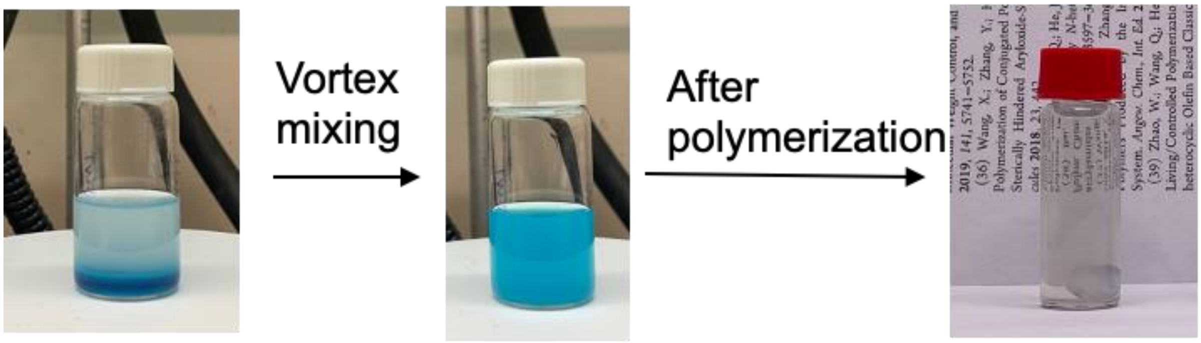

Figure S1. Digital camera image of inverse microemulsion photoATRP "cocktail" (A) before; and (B) after vortex (Table 1, Entry 1), and final resulting inverse emulsion in a one-dram vial (diameter = 15 mm) under red LED (λ_{max} = 640 nm, 25 mW cm^{-2}). [a]Standard reaction conditions: [$OEOMA_{500}$]/[HO-EBiB]/[MB^+]/[$CuBr_2$/TPMA]/[TEOA] = 50/1/0.025/0.15/0.3, $OEOMA_{500}$/$P(EO)_2C_{18}$/$P(EO)_7C_{18}$/PEG_{400}/hexane = 0.25/0.2/0.4/0.17/5 by weight (g). [$OEOMA_{500}$] = 4 wt% to total, [$P(EO)_nC_{18}$] = 9.6 wt% to total.

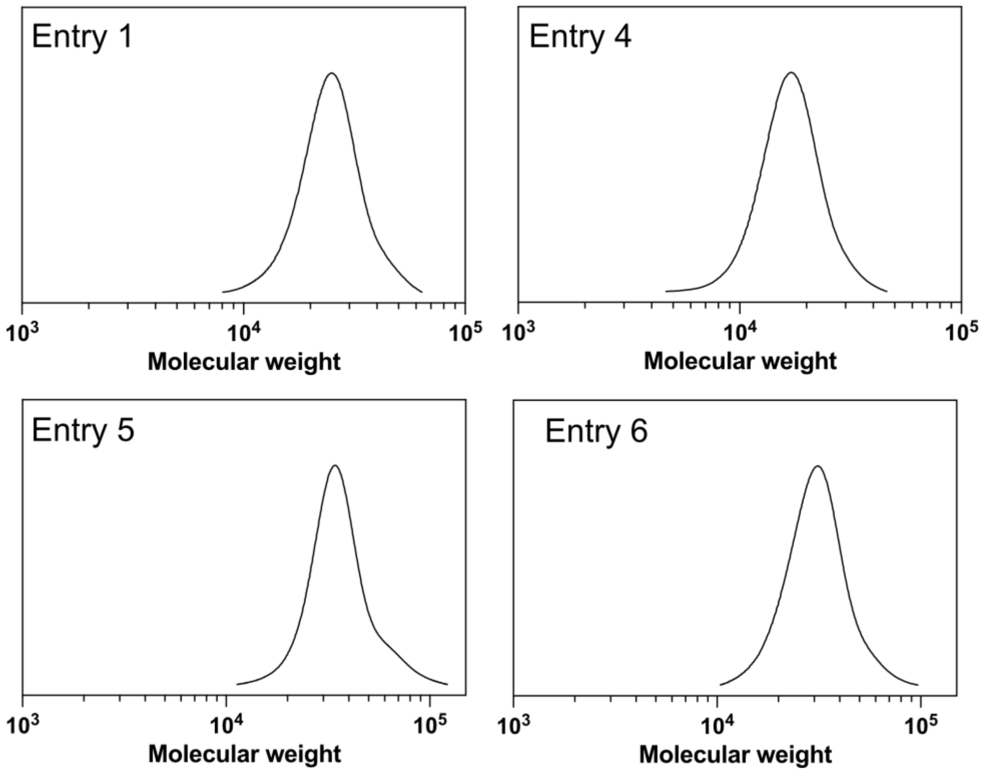

Figure S2. SEC traces for the inverse microemulsion photoATRP under reaction conditions listed in Table 1.

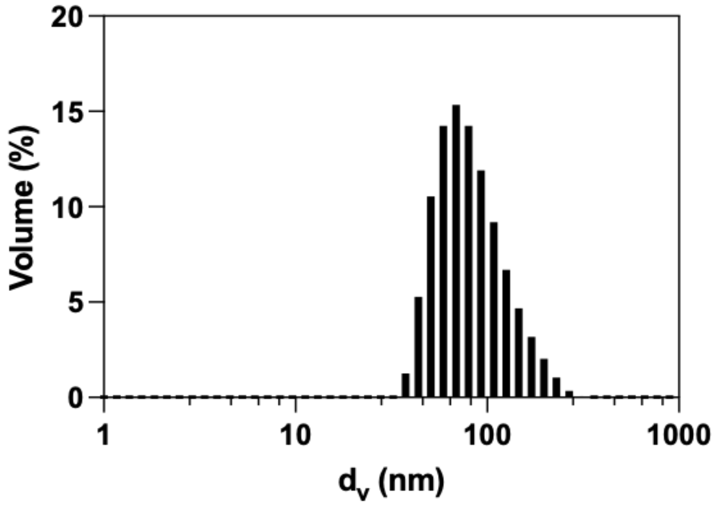

Figure S3. DLS results of the inverse microemulsion photoATRP reaction mixture under the conditions listed in Table 1 (Entry 1).

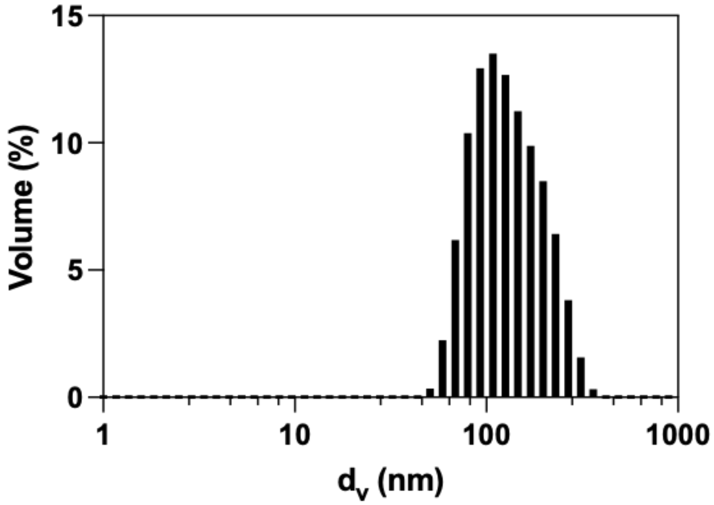

Figure S4. DLS results of inverse microemulsion photoATRP reaction mixture under the conditions listed in Table 1 (Entry 6).

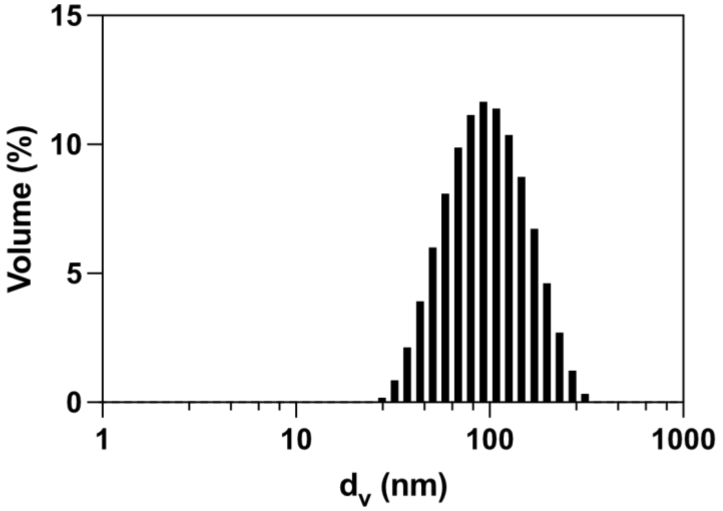

Figure S5. DLS results of pOEOMA$_{500}$-*b*-pOEOMA$_{500}$. Reaction conditions: pOEOMA$_{500}$: [OEOMA$_{500}$]/[HO-EBiB]/[MB$^+$]/[$CuBr_2$/TPMA]/[TEOA] = 50/2/0.025/0.15/0.3. pOEOMA$_{500}$-*b*- pOEOMA$_{500}$: [OEOMA$_{500}$]/[pOEOMA$_{500}$]/[MB$^+$]/[$CuBr_2$/TPMA]/[TEOA] = 50/0.4/0.025/0.15/0.3. OEOMA$_{500}$/P(EO)$_2$C$_{18}$/P(EO)$_7$C$_{18}$/PEG$_{400}$/hexane = 0.25/0.2/0.4/0.17/5 by weight (g), irradiated under red LED (640 nm, 25 mW cm^{-2}) in in a one-dram vial (5 mL, diameter = 15 mm) with stirring (700 rpm).

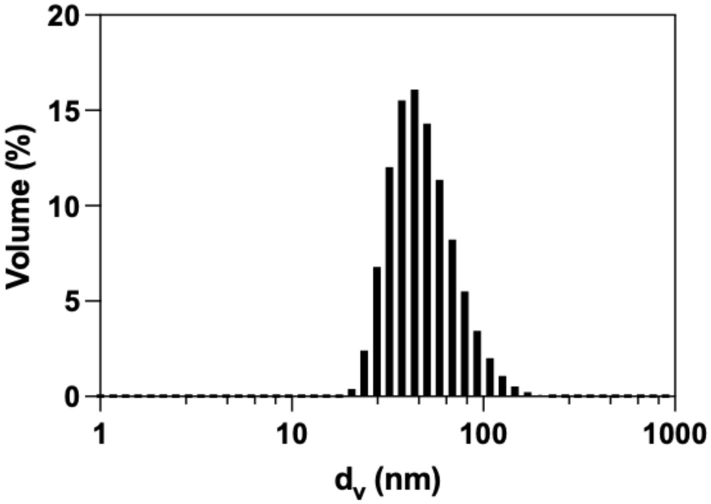

Figure S6. DLS results of PEG-b-pOEOMA$_{500}$.

Reaction conditions: [OEOMA$_{500}$]/[PEG$_{2k}$-Br]/[MB$^+$]/[$CuBr_2$/TPMA]/[TEOA] = 50/2/0.025/0.15/0.3. OEOMA$_{500}$/P(EO)$_2$C$_{18}$/P(EO)$_7$C$_{18}$/PEG$_{400}$/hexane = 0.25/0.2/0.4/0.17/5 by weight (g), irradiated under red LED (640 nm, 25 mW cm^{-2}) in in a one-dram vial (5 mL, diameter = 15 mm) with stirring (700 rpm).

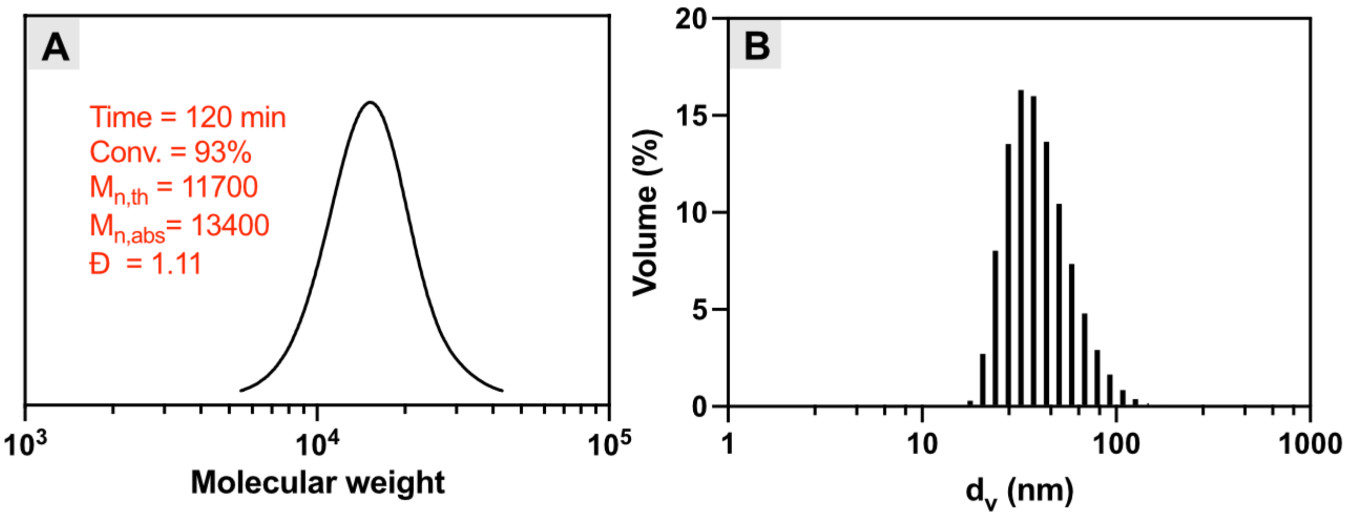

Figure S7. Temporal Control of inverse microemulsion photoATRP. (A) GPC and (B) DLS results of the resulting polymers. Reaction conditions: [$OEOMA_{500}$]/[HO-EBiB]/[MB^+]/[$CuBr_2$/TPMA]/[TEOA] = 50/2/0.025/0.15/0.3. $OEOMA_{500}$/P(EO)$_2$C$_{18}$/P(EO)$_7$C$_{18}$/PEG$_{400}$/hexane = 0.25/0.2/0.4/0.17/5 by weight (g), irradiated under red LED (640 nm, 25 mW cm^{-2}) in in a one-dram vial (5 mL, diameter = 15 mm) with stirring (700 rpm).

Table S1. Inverse microemulsion photoATRP of $OEOMA_{500}$ using different wavelength lights.[a]

Entry	Light	λ_{max} (nm)	Intensity (mW cm^{-2})	Time (h)	Conv. (%)[b]	$M_{n,th}$	$M_{n,app}$[c]	$Đ$[c]	Z_{avg} (nm)[e]
1	NIR	740	20	1	93	23300	22250	1.12	80 ± 0.6
2	Green	520	25	1	9	–	–	–	–
3	Green	520	25	4	99	25000	15100	1.12	85 ±0.3
4	Blue	460	25	1	0	–	–	–	–
5	Blue	460	25	4	99	25000	15300	1.12	91 ± 1.2
6	UV	390	25	4	99	25000	15200	1.11	85 ± 0.4

Reaction conditions: [a]Standard reaction conditions: [$OEOMA_{500}$]/[HOBiB]/[MB^+]/[$CuBr_2$/TPMA]/[TEOA] = 50/1/0.025/0.15/0.3, P(EO)$_2$C$_{18}$/P(EO)$_7$C$_{18}$/PEG$_{400}$/hexane = 0.2/0.4/0.17/5 by weight, irradiated under different light wavelength in a one-dram vial (diameter = 15 mm) with stirring. [b]Monomer conversion was determined by ^{1}H NMR with internal standard DMF. [c]Molecular weight ($M_{n,app}$) and dispersity ($Đ$) were determined by SEC analysis (DMF as eluent) calibrated to PMMA standards. [d]Absolute molecular weight ($M_{n,abs}$) was determined by Mark-Houwink calibration. [e]Average particle diameter (Z_{avg}) was determined by DLS in hexane.

References

1. Hu, X.; Szczepaniak, G.; Lewandowska-Andralojc, A.; Jeong, J.; Li, B.; Murata, H.; Yin, R.; Jazani, A. M.; Das, S. R.; Matyjaszewski, K., Red-Light-Driven Atom Transfer Radical Polymerization for High-Throughput Polymer Synthesis in Open Air. *J. Am. Chem. Soc.* **2023,** *145* (44), 24315-24327.
